# A web-based telemedicine system for low-resource settings 13 years on: insights from referrers and specialists

**DOI:** 10.3402/gha.v6i0.21465

**Published:** 2013-09-23

**Authors:** Victor Patterson, Richard Wootton

**Affiliations:** 1 Synapse Teleneurology Ltd, Belfast, UK; 2 School of Medicine, Queen's University, Belfast, UK; 3 Norwegian Centre for Integrated Care and Telemedicine, University Hospital of North Norway, Tromsø, Norway; 4 Faculty of Health Sciences, University of Tromsø, Tromsø, Norway

**Keywords:** telemedicine, web, satisfaction, developing world

## Abstract

**Background:**

One way to tackle health inequalities in resource-poor settings is to establish links between doctors and health professionals there and specialists elsewhere using web-based telemedicine. One such system run by the Swinfen Charitable Trust has been in existence for 13 years which is an unusually long time for such systems.

**Objective:**

We wanted to gain some insights into whether and how this system might be improved.

**Methods:**

We carried out a survey by questionnaire of referrers and specialists over a six months period.

**Results:**

During the study period, a total of 111 cases were referred from 35 different practitioners, of whom 24% were not doctors. Survey replies were received concerning 67 cases, a response rate of 61 per cent. Eighty-seven per cent of the responding referrers found the telemedicine advice useful, and 78% were able to follow the advice provided. As a result of the advice received, the diagnosis was changed in 22% of all cases and confirmed in a further 18 per cent. Patient management was changed in 33 per cent. There was no substantial difference between doctors and non-doctors. During the study period, the 111 cases were responded to by 148 specialists, from whom 108 replies to the questionnaire were received, a response rate of 73 per cent. About half of the specialists (47%) felt that their advice had improved the management of the patients. There were 62 cases where it was possible to match up the opinions of the referrer and the consultants about the value of a specific teleconsultation. In 34 cases (55%) the referrers and specialists agreed about the value. However, in 28 cases (45%) they did not: specialists markedly underestimated the value of a consultation compared to referrers. Both referrers and specialist were extremely positive about the system which appears to be working well. Minor changes such as a clearer referral template and an improved web interface for specialists may improve it.

Telemedicine systems have been employed in low-resource settings for about 20 years, but those that have stood the test of time are unusual. The system set up by the Swinfen Charitable Trust (SCT) in 1999 is one such system; it provides medical advice to doctors and other health professionals in low-resource countries using a network of specialists from around the world. We have surveyed its referrers and specialists to investigate whether there is scope for improvement.

Formal evidence supporting the value of such systems is still rather weak (1). In particular, quantitative data about patient outcomes are limited. This is probably not due to a lack of interest from the research community, but because of the serious practical difficulties surrounding the follow-up of patients in low-resource settings. It is not uncommon, for example, for patients to present at rural facilities, have their treatment initiated (with or without telemedicine advice) and then to simply vanish from the healthcare system.

In the context of telemedicine in low-resource settings, there have been few studies of user satisfaction. Indeed, there only appears to have been one study of patient satisfaction (2), which contrasts with the situation in the industrialised world, where patient satisfaction with telemedicine has been much studied (3). In these latter studies, as in that of Heinzelman et al. (2), the majority of patients report general satisfaction with telemedicine.

Much of the information about the value of telemedicine in low-resource settings has been obtained from surveys of the referring doctors involved. Zolfo et al. surveyed user satisfaction with the HIV/AIDS support network operated by the Institute of Tropical Medicine in Antwerp (4). Users reported that the telemedicine service had influenced the management of the patients in 90% of the cases and that the advice had been beneficial for several reasons, particularly for establishing the diagnosis (52%) (5).

Wootton et al. surveyed referring doctors using the Swinfen telemedicine network in 2003. The results indicated that the advice provided was used by 93% of the respondents, and 79% found it helpful. Over half (53%) of all respondents indicated that the advice provided changed the management of the patient (6).

There appear to have been few other published reports of the opinions of users of telemedicine networks in low-resource settings and almost nothing is known about the views of the specialists who provide advice in these networks. The opinions of a neurologist who dealt with neurology referrals by email were recorded in two separate situations (7, 8). The present study was therefore conducted to obtain the views of both referrers and specialists in a longstanding telemedicine network.

## Methods

We surveyed the doctors and other health professionals who had referred cases through the web-based messaging system during a 6-month period starting in June 2012. A short survey was sent by email, asking nine questions about the value of each teleconsultation (see Appendix).

In addition, the specialists who had responded to each case were sent a similar short survey by email, asking 10 questions about the value of the particular teleconsultation (see Appendix).

The surveys were sent approximately 10 days after the initial referral, to allow time for the referring doctor to consider the advice provided and to implement it if appropriate.

## Results

**Fig.1:**
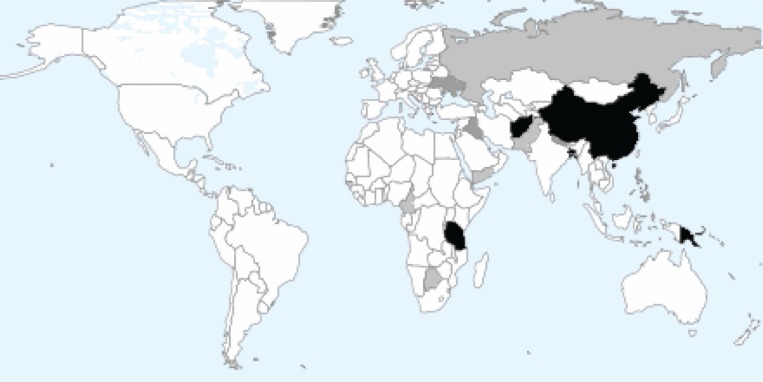
Referring countries. Black shading >10 referrals; Dark grey 5–9 referrals; Light grey <5 referrals (map courtesy of Aneki.com, see http://www.aneki.com/map.php).

During the study period, a total of 111 cases were referred from 35 different referrers. The median interval between the initial referral and the surveys being sent to referrers was 11 days. Replies were received concerning 67 cases, a response rate of 61%. The responses were from 25 different referrers who came from 15 different countries (Fig. 1).

The median interval between the initial referral and the surveys being sent to the specialists was 10 days. The 111 cases were responded to by 148 specialists from whom 108 replies to the questionnaire were received, with a response rate of 73%. These responses concerned 88 cases and came from 54 individual specialists from 9 different countries (Table 1). The main area of expertise of the responding specialists is shown in Table 2.

**Table 1:** Country of origin of the specialist replies

	Number
Australia	10
Austria	2
Bangladesh	1
Canada	5
Germany	3
Ireland	2
New Zealand	6
UK	52
USA	27
Total	108

**Table 2:** Main area of expertise of the responding specialists

	Number		Number
Paediatrics	37		
		Cardiology	3
		Dermatology	2
		Endocrinology	1
		Gastroenterology	1
		General	5
		Neonatal	8
		Neurology	3
		Neurosurgery	2
		Oncology	3
		Orthopaedics	2
		Radiology	3
		Renal	1
		Respiratory	1
		Surgery	2
Medical specialties	35		
		Dermatology	7
		Endocrinology	3
		Gastroenterology	2
		Genetics	3
		Haematology	1
		Infectious diseases	2
		Neurology	8
		Oncology	1
		Renal	1
		Respiratory	6
		Tropical diseases	1
Surgery	22		
		ENT	1
		Oncology	1
		Ophthalmic	1
		Orthopaedics	11
		Plastic	5
		Urology	3
Radiology	7		
		General	2
		Neuroradiology	5
Pathology	4		
		Haematology	4
Obstetrics/gynaecology	3		
		General	1
		Oncology	1
		Ultrasound	1
Total	108		108

### Referrers

Eighty-seven per cent of the responding referrers found the telemedicine advice useful, and 78% were able to follow the advice provided (Table 3). The advice could not be followed in 16% of cases; three-quarters of those who gave a reason said that the necessary tests or procedures were not available locally.

**Table 3:** Response to referrers’ yes/no questions (67 replies)

	Yes (%)	No (%)
Were you able to follow the advice given?	78	16
Did you find the advice helpful?	87	6
Was there any educational benefit to you in the reply?	75	16
Was there any cost saving resulting from this consultation?	46	30
Is the patient likely to be available for follow-up in 6 months time?	60	10
Would you be happy to use the service again?	88	0

As a result of the advice received by email, the diagnosis was changed in 22% of cases and confirmed in a further 18%. Patient management was changed in 33%.

Most of the referrers who responded were doctors (76%), but some were non-doctors (24%), that is, nurses or physiotherapists. A separate analysis of the doctors’ and non-doctors’ responses to these questions showed no substantial differences.

Thirty-six referrers replied to the question, ‘What is the outcome for the patient?;’ 44% felt there was improvement, 56% that they were the same and none felt that they were worse.

The majority of free-text responses about the case specifically or the service generally were positive comments (91%). The free-text responses are summarised in Table 4.

**Table 4:** Referrers’ free-text comments (total number of replies, 35)

	Number
Positive comments	32
Difficulty in uploading Dicom images	2
Possibility of another opinion if the first one misses the point	1

According to the referrers, approximately half of the teleconsultations produced cost savings. The cost saving resulting from the consultations accrued to the patient and their family in 27 instances (59%), to the hospital or clinic in 10 (22%) and to both in 7 (15%). Details are shown in Table 5.

**Table 5:** Savings made as a result of consultations (total number of replies, 22)

	Number
Fewer doctor visits	8
Saved in-country travel	5
Saved international travel	4
Avoided investigations	4
Avoided surgery	1
Fewer drugs needed	1

### Specialists

About half of the specialists (47%) felt that their advice had improved the management of the patient (Table 6).

**Table 6:** Specialists’ responses to yes/no questions (total number of replies, 108)

	Yes (%)	No (%)
Was the information supplied by the referrer (including any images) adequate?	70	30
Was the question asked by the referrer clear?	92	7
Was it difficult to find the time required to answer this case?	6	95
Do you think the advice you provided will improve the management of this patient?	47	9
Would you like to receive follow-up information about this patient?	83	16
Do you have any concerns about the telemedicine process? (e.g. liability, data security)	19	81
Did the consultation have any value for you personally?	31	65
Are you happy to provide more consultations for the SCT in the future?	95	0

SCT, Swinfen Charitable Trust.

About one-third of responding consultants (30%) thought that the referral information was not adequate, mostly because of a lack of relevant clinical information or imaging results (Table 7). When analysed separately for radiologists (*n*=10) and clinicians (*n*=96), there was no difference in the adequacy of the information supplied.

**Table 7:** Reasons for the specialists feeling that the referral information was inadequate (total number of replies, 35)

	Number
Inadequate clinical information	17
Inadequate images or test results	7
Lack of information about referrer's local circumstances	4
Difficulty viewing attached images or documents	2
Case too complicated for the system	2
Case records mixed-up	1
Uncertainty about referrer's English	1

The majority of free-text responses about the case specifically or the service generally were positive comments (60%). The free-text responses are summarised in Table 8.

**Table 8:** Specialists’ free-text responses (total number of replies, 57)

	Number
Supportive and complimentary	34
More feedback	5
Information on local services and specialists	4
Better referrals	4
Produce annual report	3
Liability and disclaimer	2
Second opinion abuse	1
Develop private practice	1
Concern about misunderstanding due to poor English	1
Referrers should obtain consent to use photos for teaching	1
Develop a chronic illness system	1
Develop a phone app	1

There were 62 cases where it was possible to relate the opinions of the referrer and the consultants about the value of a specific teleconsultation. In 34 cases (55%), the referrers and specialists agreed about the value. However, in 28 cases (45%) they did not: specialists markedly underestimated the value of a consultation compared to referrers, as shown in Table 9. Also, in 17 of these shared cases the specialists judged the information as inadequate, yet the referrers found the specialist's advice helpful in 76% of them. Furthermore, it changed diagnosis in 29% and management in 35% of these cases.

**Table 9:** Opinions of the referrers and specialists about the value of individual teleconsultations (responses to the question: Was the advice useful?).

		Referrers	
		
		Yes	No
Specialists	Yes	29	0
	No	28	5

The values shown are based on 62 cases for which both referrer and specialist opinions were available.

## Discussion

The tone of the responses from both the referrers to the system and the replying specialists was overwhelmingly positive and supportive of this telemedicine network. Responders were unanimous in wishing to use the system again (referrers) or continuing to provide expert advice (specialists). The free-text comments were also very supportive – many specialists were pleased to have this opportunity to assist colleagues in resource-constrained settings and referrers felt supported by having someone to call on easily for advice. There is therefore a mutual beneficence in the system. This confirms the findings from the previous survey (6) and the reports of others (2, 4, 5).

Over the last few years, the system has begun to take referrals from other health professionals who are responsible for patients in remote areas, that is, nurses, physiotherapists, and health workers. A separate analysis of the responses from the latter did not show much difference from the responses of the doctors. This is important, because if the medical needs of people in the developing world are to be improved then health professionals other than doctors will have to play a major role.

There is clear evidence of perceived benefits to patients as a result of this telemedicine system. Referrers thought that about a quarter of patients were better after the consultation and that the consultation helped over 90% of cases. This accords with the rather limited information available from patient follow-up in Papua New Guinea (9). Diagnosis was changed in 22% of referred cases and patient management in 33%, which would be considered as excellent outcomes for face-to-face consultations in the industrialised world. Even where the consultation did not change diagnosis or management, referrers felt that it was beneficial to them in 31 out of 36 cases (86%). Interestingly, specialists markedly underestimated the benefit of their consultations as shown in Table 9. This has been noted previously in a study of email teleneurology (7). The likely explanation is that referrers feel considerably empowered by specialist advice even though the specialists do not think that they offered anything new.

There was little change in these figures from the previous study of this network in 2004 (6) when 79% of referrers thought that the advice was helpful and that it changed management in 53%.

The responders also reported clear benefits in terms of cost savings, particularly to the patients. Reduced need for travel, further medical consultations and investigations implies good diagnosis and clear management, which can be done using telemedicine.

Educational benefits accrued to 82% of referrers and 33% of specialists. One surprising finding concerned the potential for follow-up: while it had been assumed that follow-up of patients referred to this network would be difficult, 60% of referrers felt that the patient would be available for review in 6 months time. This is particularly relevant as it was the overwhelming wish of specialists to have more follow-up information on the patients they gave an opinion on.

The main problem highlighted in the survey of specialists was that the information provided by the referrer was inadequate to provide an opinion in 30% of referrals due to a lack of either proper clinical information or test results. At present, the referrer enters the referral details in free-text, and it may be that a more structured clinical pro forma might improve this. Poor-quality images were identified as a problem in 7 out of the 35 cases where the referral information was inadequate. The quality of the photographs taken by doctors in the field remains a problem for telemedicine networks (10). Otherwise, there were relatively few technical difficulties with the use of the secure web platform. Information about the local circumstances of each referrer was available on the system, but perhaps not displayed prominently enough.

The other issue raised by about one-fifth of the specialists was concern about their legal liability in providing these remote consultations. In general, the bulk of the liability will rest with the referrer, but this is not an area of certainty and there will be variations both between and within countries on how liability for specialists dealing with remote consultations is handled by the providers of medical insurance. Fortunately, there continues to be an absence of case law on this topic.

This study had certain limitations. Although the response rate to the survey was high, as judged by response rates in postal surveys, it was not 100%, and we have no information about the views of non-responders. A central weakness is that referring doctors who have used the service are almost bound to say positive things about it. In future, we would be interested in knowing the opinions of potential referring doctors who have not used the service.

The Swinfen telemedicine system is one of the longest running of such systems and it is clearly working well. Two factors emerge from this study that might explain this. One is the clear benefit to patients and another is the mutual beneficence to referrers and specialists, where the former have easy access to expert medical advice and the latter have the opportunity to help colleagues in the developing world without leaving their offices. Another feature is the distinctly light touch with which the telemedicine work is coordinated; this makes using the system remarkably simple for referrers and specialists, and for the latter offers a stark contrast to their often bureaucratic day-to-day work in hospitals and universities. This study highlights some minor areas which are potentially remediable: a better information template, a clearer web interface for specialists and the opportunity for follow-up information.

When systems are not broken they do not need to be fixed. The small changes suggested by this study should not affect this system's continued operation.

## Appendix. Questionnaires

**Referrers’ survey**

1. Were you able to follow the advice given?

  -Yes

  -No

If NO, could you explain briefly why not ….

2. Did you find the advice helpful?

  -Yes

  -No

If YES, did it- (tick any that apply)

  -Change your diagnosis

  -Change your management

  -Improve the patient's symptoms

  -Improve function

  -Any other reason? Please specify ….

3. What is the outcome for the patient?

  -Better

  -Worse

  -The same

  -Don't know

4. Was there any educational benefit to you in the reply?

  -Yes

  -No

5. Was there any cost saving resulting from this consultation? (tick any that apply)

  -Yes – saving for the patient/family

  -Yes – saving for the hospital/clinic

  -No

  -Don't know

If YES, please explain briefly ….

6. Is the patient likely to be available for follow-up in 6 months time?

  -Yes

  -No

  -Don't know

7. Please add any other comments about this case specifically ….

8. Would you be happy to use the service again?

  -Yes

  -No

9. Please add any other comments about the service generally ….

** Specialists’ survey**

1. Was the information supplied by the referrer (including any images) adequate?

  -Yes

  -No

If NO, could you explain briefly why not ….

2. Was the question asked by the referrer clear?

  -Yes

  -No

3. Was it difficult to find the time required to answer this case?

  -Yes

  -No

4. Do you think the advice you provided will improve the management of this patient?

  -Yes

  -No

  -Don't know

5. Would you like to receive follow-up information about this patient?

  -Yes

  -No

6. Do you have any concerns about the telemedicine process? (e.g. liability, data security)

  -Yes

  -No

If YES, could you explain briefly ….

7. Did the consultation have any value for you personally? (e.g. images that can be used for teaching [with the permission of the referrer])

  -Yes

  -No

If YES, could you explain briefly ….

8. Please add any other comments about this case specifically ….

9. Are you happy to provide more consultations for the SCT in the future?

  -Yes

  -No

10. Please add any other comments about the service generally ….

## References

[1] Wootton R, Geissbuhler A, Jethwani K, Kovarik C, Person DA, Vladzymyrskyy A (2012). Long-running telemedicine networks delivering humanitarian services: experience, performance and scientific output. Bull World Health Organ.

[2] Heinzelman PJ, Jacques G, Kvedar JC (2005). Telemedicine by email in remote Cambodia. J Telemed Telecare.

[3] Whitten P, Love B (2005). Patient and provider satisfaction with the use of telemedicine: overview and rationale for cautious enthusiasm. J Postgrad Med.

[4] Zolfo M, Bateganya MH, Adetifa IM, Colebunders R, Lynen L (2011). A telemedicine service for HIV/AIDS physicians working in developing countries. J Telemed Telecare.

[5] Zolfo M, Lorent N, Bateganya M, Kiyan C, Lequarré F, Koole O Telemedicine in HIV/AIDS care: a users’ satisfaction survey.

[6] Wootton R, Youngberry K, Swinfen P, Swinfen R (2004). Prospective case review of a global e-health system for doctors in developing countries. J Telemed Telecare.

[7] Patterson V, Hoque F, Vassallo D, Farquharson Roberts M, Swinfen P, Swinfen R (2001). Store-and-forward teleneurology in developing countries. J Telemed Telecare.

[8] Patterson V, Swinfen P, Swinfen R, Azzo E, Taha H, Wootton R (2007). Supporting hospital doctors in the Middle East by email telemedicine: something the industrialized world can do to help. J Med Internet Res.

[9] Wootton R, Menzies J, Ferguson P (2009). Follow-up data for patients managed by store and forward telemedicine in developing countries. J Telemed Telecare.

[10] Jakowenko J, Wootton R (2006). An analysis of the images attached to referral messages in an email-based telemedicine system for developing countries. J Telemed Telecare.

